# Pro-inflammatory Cytokines Alter the Immunopeptidome Landscape by Modulation of HLA-B Expression

**DOI:** 10.3389/fimmu.2019.00141

**Published:** 2019-02-18

**Authors:** Aaron Javitt, Eilon Barnea, Matthias P. Kramer, Hila Wolf-Levy, Yishai Levin, Arie Admon, Yifat Merbl

**Affiliations:** ^1^Department of Immunology, Weizmann Institute of Science Rehovot, Israel; ^2^Faculty of Biology, Technion-Israel Institute of Technology, Haifa, Israel; ^3^The Nancy and Stephen Grand Israel National Center for Personalized Medicine, de Botton Institute for Protein Profiling, Weizmann Institute of Science, Rehovot, Israel

**Keywords:** immunopeptidomics, cancer, proteomics, bioinformatics, inflammation, HLA

## Abstract

Antigen presentation on HLA molecules is a major mechanism by which the immune system monitors self and non-self-recognition. Importantly, HLA-I presentation has gained much attention through its role in eliciting anti-tumor immunity. Several determinants controlling the peptides presented on HLA have been uncovered, mainly through the study of model substrates and large-scale immunopeptidome analyses. These determinants include the relative abundances of proteins in the cell, the stability or turnover rate of these proteins and the binding affinities of a given peptide to the HLA haplotypes found in a cell. However, the regulatory principles involved in selection and regulation of specific antigens in response to tumor pro-inflammatory signals remain largely unknown. Here, we chose to examine the effect that TNFα and IFNγ stimulation may exert on the immunopeptidome landscape of lung cancer cells. We show that the expression of many of the proteins involved in the class I antigen presentation pathway are changed by pro-inflammatory cytokines. Further, we could show that increased expression of the HLA-B allomorph drives a significant change in HLA-bound antigens, independently of the significant changes observed in the cellular proteome. Finally, we observed increased HLA-B levels in correlation with tumor infiltration across the TCGA lung cancer cohorts. Taken together, our results suggest that the immunopeptidome landscape should be examined in the context of anti-tumor immunity whereby signals in the microenvironment may be critical in shaping and modulating this important aspect of host-tumor interactions.

## Introduction

Antigen processing and presentation is a major cellular mechanism through which cells are monitored by the immune system. Specifically, the immune system can identify diseased cells through peptides, that are presented on class I and II Human Leukocyte Antigen (HLA) (1). The repertoire of presented peptides is defined through a combination of determinants, including the proteins found in the cell, how they are processed into peptides by proteasomes (MHCI) or lysosomes (MHCII) and further bound by HLA molecules and transported to the cell surface (2–7). Numerous studied have analyzed the peptides presented on HLA in many physiological states. However, our knowledge about the underlying molecular mechanisms by which cells modulate their presented repertoire in response to an altered cellular environment or different stimuli remains largely unknown.

Previous studies, characterizing peptides that are presented on HLA, found that these peptides originate from proteins throughout the cell (8–10). With technological advancements of mass spectrometry, higher coverage of the peptidome presented on HLA, or immunopeptidome, has been achieved. These advancements allowed for identification of thousands of peptides per biological sample, thereby revealing the immunopeptidome landscape. Recent, studies have revealed that up to 40% of the immunopeptidome differs between cell types, elucidating the potential complexity of the regulation of antigen presentation in physiological systems (11).

The determinants affecting and regulating antigen presentation may be generally divided into two categories, namely cis or trans-regulation. The first (cis) relies on the properties of the protein that is being cleaved and presented (e.g., peptide sequence, expression level) and the second (trans) relies on the enzymatic machinery and cellular proteins that are involved in its cleavage, HLA binding and shuttling to the cell surface. Indeed, numerous cis-factors were already shown to play a role in determining the presented peptidome, including transcript abundance, protein abundance, protein length and protein degradation rate (12–16). Nevertheless, there is no clear consensus on the specific contribution of each of these factors to the selection of peptides and their presentation. Importantly, the relative contribution of each factor may differ between biological states. For example, the presentation of viral antigens has been linked to the translation rate of viral proteins, not their degradation rate (17). During cellular infection, upon development of cancer or in the course of autoimmune diseases the proteome of the cell is altered. This is due to the introduction of novel exogenous proteins, changes in protein expression levels or mutations that arise in endogenous cellular proteins through protein translation (18–20). The relative frequencies with which proteins are degraded, or protein half-lives, change in various disease states (21, 22) and these factors influence peptide selection and presentation.

As mentioned above, some determinants contribute in trans by modulating the expression and activity of the various components of the antigen processing and presentation machinery, influencing the presented peptidome. In the case of class I presentation these include the proteasome, Transporter Associated with Antigen Presentation (TAP), cellular peptidases and the HLA complex itself (23–29). In particular, the specific haplotypes of HLA have a strong effect on the repertoire of presented peptides. Each HLA variant has different binding constraints that determine a biochemical motif of the peptides presented on the complex (30, 31). Greater diversity of HLA haplotypes leads to a larger and more diverse presented peptidome (12). These constraints have been studied through the comparison of different cell lines or tissues, which have different haplotypes, as well as direct biochemical studies on individual haplotypes. Additionally, the binding properties form the basis of algorithms for predicting the peptides, which could be bound by a given HLA molecule (32).

Importantly, most of the studies conducted thus far focused on cells grown *in vitro*, due to the large quantities of material needed to analyze the HLA peptidome. Less attention has been given to elucidation of the plasticity of the presented peptidome or to how the peptidome is modulated by changes in the cellular proteome. For example, following treatment with the mTOR inhibitor rapamycin, the presented peptidome changed, corresponding to the change in the cellular proteome and proteasome activity (33). In addition, proteasome cleavage patterns contribute to the immunopeptidome. It was previously shown that pro-inflammatory cytokines induce immunoproteasomes, which generate peptides that are more hydrophobic in nature and are more likely to be presented by HLA molecules (34). Immunoproteasomes have two chymotrypsin-like catalytic subunits, in contrast to the single subunit in constitutive proteasomes (35). Accordingly, induction of immunoproteasomes by IFNγ has been shown to increase the presentation of long peptides with chymotryptic-like cleavage patterns (36). Further it was shown that upon deletion of two subunits of the immunoproteasome, MECL1 and LMP7, the presented peptidome decreased in depth and diversity (11). In the case of a specific antigen, NY-ESO-1, IFNγ mediated induction of the immunoproteasome changed the peptides presented and the resulting T cell response (37). In addition to their effect on immunoproteasomes, TNFα and IFNγ were also shown to increase the amount of HLA molecules on the cell surface, and specifically increase the amount of HLA-B as there are two interferon response elements upstream of the HLA-B locus, whereas the HLA-A and -C locus only contain one (38–41). Recently, the relative changes in haplotype expression on the immunopeptidome, in response to IFNγ-induced upregulation of HLA expression, was analyzed by Komov et al. on MCF-7 cells (42).

Here, we examined the effect of TNFα and IFNγ stimulation on the immunopeptidome. The combination of the two cytokines work synergistically to modulate the levels of the antigen processing and presentation machinery (43–47) and may reflect the pro-inflammatory conditions in a tumor microenvironment (TME). We used mass spectrometry (MS) based measurements of both the cellular proteins (proteome) and the HLA class I peptidome of the lung epithelial cell line, A549, which was previously shown to upregulate immunoproteasome expression in response to inflammatory signal (48). Our analysis revealed a mechanism of direct modulation of the peptidome through an inflammatory signal, which alters the relative expression of the HLA haplotypes. Our results suggest that the microenvironment of cells may significantly affect the landscape of the presented peptidome via modulation of the expression of specific haplotypes.

## Results

### TNFα and IFNγ Signaling Increases Presentation of MHC I Peptides

**Figure 1:**
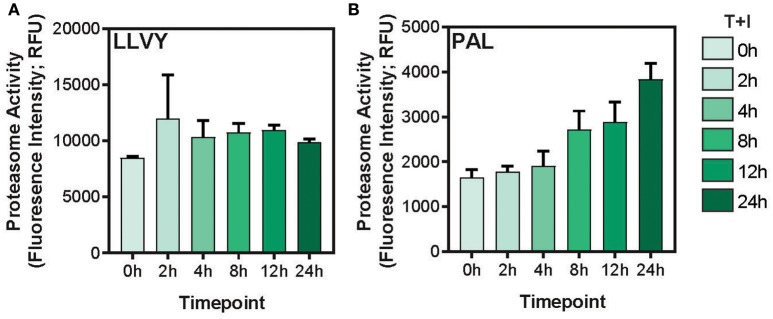
Immunoproteasome activity is induced by TNFα and IFNγ stimulation. **(A)** The Suc-LLVY-AMC constitutive proteasome substrate or **(B)** the Ac-PAL-AMC immunoproteasome substrate were incubated with lysates after treatment with TNFα and IFNγ for the indicated times (0–24 h). The final fluorescence measurement in relative fluorescence units (Fluorescence Intensity; RFU) following 3.5 h of incubation with the substrate is plotted (error bars indicate standard deviation).

**Figure 2:**
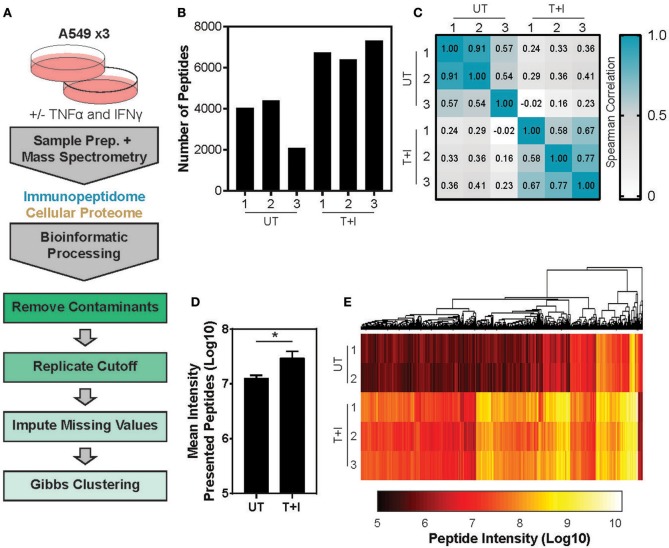
Analyzing the TNFα and IFNγ stimulated presented peptidome. **(A)** Experimental and informatics workflow includes stimulation of A549 cells with TNFα and IFNγ for 24 h, harvesting samples which were subject to MS-analysis of both the peptides presented on MHC I (immunopeptidome) and total cellular lysates (Cellular Proteome). Thousands of peptides were identified, common contaminates were removed as well as peptides which were not identified in at least 2 of 3 replicates (replicates). Missing values were imputed to randomly chosen values from a normal distribution and the resulting peptides were subject to Gibbs Clustering. **(B)** The number of unique peptides identified in the presented peptidome of each of the 3 triplicates stimulated with TNFα and IFNγ (T+I) or left untreated (UT). **(C)** Spearman Rho of the peptide intensities from each pairwise combination of samples. **(D)** The mean presented peptide intensity (Log10) in the unstimulated (UT) and stimulated (T+I) samples (^*^p = 0.0215; error bars indicate SD). **(E)** Supervised clustering of peptide intensities with and without stimulation of cells with TNFα + IFNγ (T+I). Only peptides which significantly differed in abundance between the two conditions are displayed (city-block distance function on Log10 transformed intensity). Significance is determined as *p* ≤ 0.05 (Benjamini-Hochberg FDR corrected *P*-values, student's *t*-test).

This study utilized A549 cells, a KRAS-driven cancer cell line derived from alveolar epithelial cells. To induce an inflammatory-like response *in-vitro*, the cells were stimulated with TNFα and IFNγ (hereby referred to as “stimulation”). As previously shown (49) upon stimulation, the surface expression of HLA was increased, as detected by flow cytometry analysis (Supplementary Figures 1A,B). Furthermore, stimulation with pro-inflammatory cytokines also induced a change in proteasome activity in the cells. Following stimulation, the level of the immunoproteasome catalytic subunits, β2i and β5i, was increased as detected by western blot (Supplementary Figure 1C). This was accompanied by a slight decrease in the level of the constitutive catalytic subunit β5 (Supplementary Figure 1C). To examine whether the pro-inflammatory cytokines changed the proteasome activity, we tested cleavage of two fluorogenic substrates, Ac-PAL-AMC and Suc-LLVY-AMC that are used to assess the catalytic activity of the immuno- and constitutive proteasome, respectively (50). When supplemented into functional extracts, these peptides are released and the fluorescence of the AMC moiety is measured. While the constitutive proteasome activity did not increase (Figure 1A), an increase in immunoproteasome-associated activity after TNFα and IFNγ stimulation was observed (Figure 1B). Furthermore, while the duration of stimulation did not significantly increase the activity of the constitutive proteasome Figure 1A; one way ANOVA: [*F*(5, 12) = 1.372, *p* = 0.3], the activity of the immunoproteasome significantly increased Figure 1B; one way ANOVA: [*F*(5, 12) = 19.84, *p* < 0.0001]. Finally, we confirmed that there was no significant decrease in cellular viability following 24 hours of cytokine stimulation (Supplementary Figure 1D). Given that there are dramatic changes in the MHC peptide processing and presentation pathway upon cytokine signaling, we set out to profile the immunopeptidome landscape after 24 h of stimulation (Figure 2A). A549 cells were stimulated with TNFα and IFNγ (denoted as T+I) or left untreated (UT) and HLA-bound peptides were isolated and analyzed by LC-MS/MS and the MaxQuant software tool (51) as previously described (42). Following filtering and processing steps (detailed in the materials and methods), we identified an average of 3,444 peptides in the untreated samples and 6,582 peptides in the treated samples (Figure 2B). Biological replicates showed 0.56–0.91 spearman correlation (Figure 2C), while the stimulated and untreated samples did not correlate with one another, rho = −0.02 to 0.41 (Figure 2C and Supplementary Figure 2A). Due to the low number of peptides identified in the third triplicate of untreated cells (UT-3), as well as the poor correlation to the other samples in the triplicate (Figures 2B,C), the sample was excluded from the analysis (see materials and methods). However, we confirmed the validity of our conclusions was not affected by this exclusion (Supplementary Figures 3A–C). Importantly, we found a significant increase in peptide abundance after cytokine stimulation (Figure 2D; ^*^ *p* = 0.0215). Of the peptides which significantly changed in abundance, most were increased by the stimulation (Figure 2E).

### The Presented Peptidome Alters Significantly in Response to TNFα and IFNγ Stimulation

In conjunction with the MHC profiling, the proteome of whole cell extracts (WCE) from the same culture and treatment condition were analyzed using bottom-up proteomics to examine the “cellular proteome”. We identified between 4,062 and 4,305 proteins per replicate. As expected, we found numerous proteins whose abundance increased upon stimulation and which are known to be involved in the response to IFNγ, such as STAT1 (52, 53) (Supplementary Figure 4A; Supplementary Table 1). Furthermore, many of the known interferon inducible proteins increased in abundance (e.g., IFIT1, IFIT2, and IFIT3). We then examined which pathways were changed as a result of the cytokine stimulation using PANTHER (54). As would be expected, the most enriched pathway was that of Antigen Processing and Presentation (Supplementary Figure 4B; GO: 0019882). Likewise, we identified response to IFNγ as another significantly enriched pathway (GO: 0034341). However, the proteins representing the HLA peptides identified in the immunopeptidome analysis were not significantly enriched for specific biological processes or pathways.

**Figure 3:**
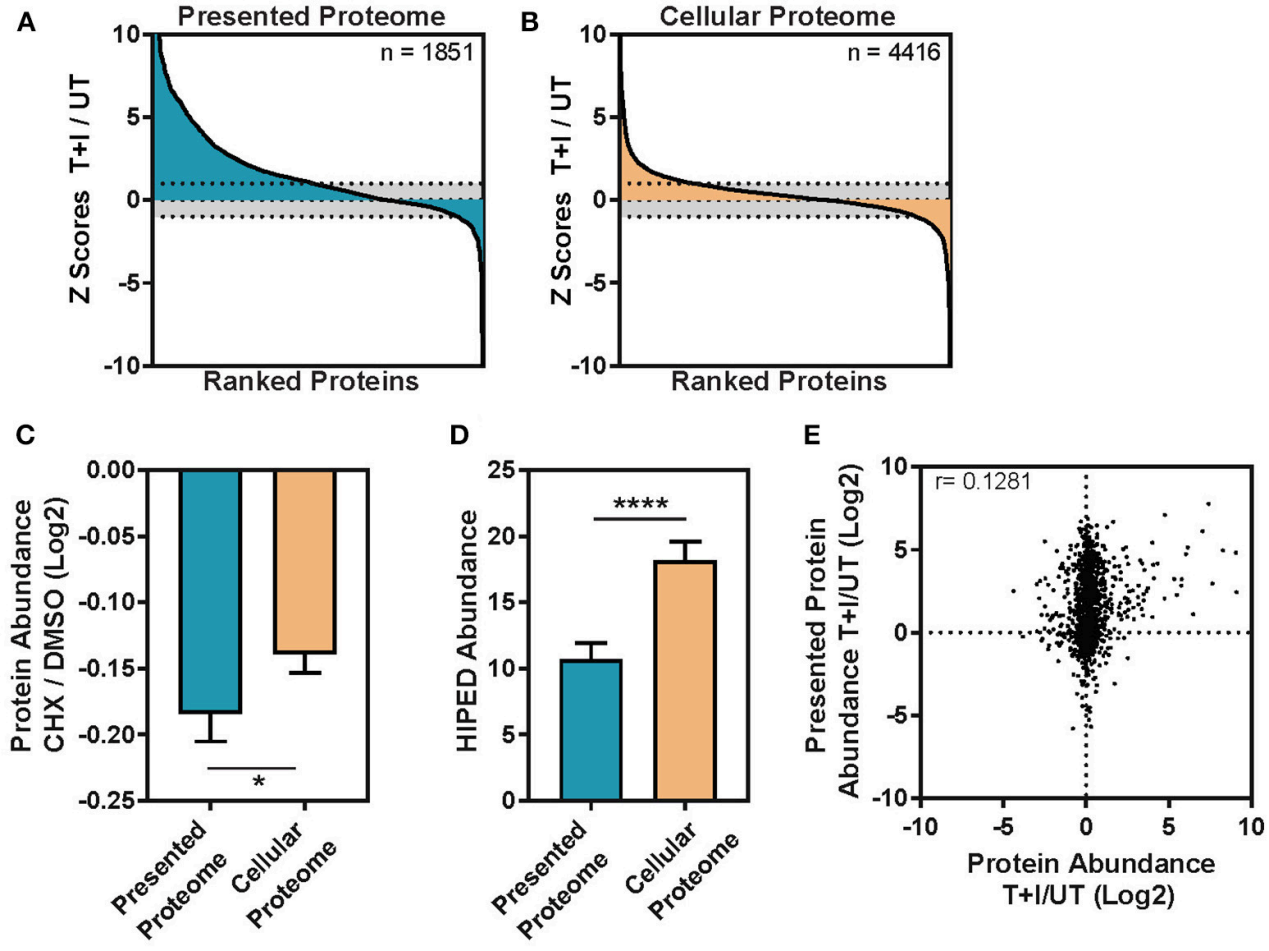
The change induced in the TNFα and IFNγ stimulated presented peptidome is not driven by the abundance change in the cellular proteome. **(A)** The immunopeptides were inferred to parent proteins (Presented Proteome) or **(B)** the proteins in the cellular lysate (Cellular Proteome) and then ranked based on the change in intensity between TNFα and IFNγ stimulated and unstimulated cells. Each protein was assigned a standard score (*Z* score) based on the magnitude of the deviation between the stimulated and unstimulated protein abundances. Scores >1 standard deviation were considered significant (dotted line = 1 sd.). **(C)** Protein turnover ratios were taken from Larance et al. (55) who measured the decrease in protein abundance following treatment with the protein synthesis inhibitor cycloheximide (CHX/DMSO, log2 transformed). The turnover ratio was here applied to the subset of proteins which contain presented peptides (presented proteome) and those which are only found in the cellular lysate (cellular proteome). Error bars indicate confidence intervals on the mean (Mann-Whitney ^*^p = 0.0182) **(D)** Protein abundance was inferred from the GeneCards Suite Human Integrated Protein Expression Database (HIPED) (56) and applied to the subset of proteins which contain presented peptides (Presented proteome) and those which are only found in the cellular lysate (cellular proteome). Error bars indicate confidence intervals on the mean (Mann-Whitney ^****^p < 0.0001). **(E)** The ratio of cellular protein abundance between stimulation with TNFα + IFNγ (T+I) and unstimulated (UT) is plotted against the ratio in intensity for the presentation of each protein (Log2 transformed ratios, Pearson correlation (r) is displayed on the graph, *n* = 1,239).

When we compared the proteins identified in the WCE and immunopeptidomics we found 25% that were detected in both, 63% that were detected only in the cellular lysate and 12% that were exclusive to the immunopeptidomics (Supplementary Figure 5A). The fraction of proteins identified solely in the immunopeptidomics pool may represent proteins that are characteristically difficult to identify from WCE, such as low-abundance, high-turnover proteins or trans-membrane proteins. Interestingly, the immunopeptidome changed more dramatically upon cytokine stimulation than the cellular proteome (Figures 3A,B). By examining the change in abundance for each protein detected, we found that almost 50% of the proteins containing immunopeptides (denoted as parent proteins) significantly increased in abundance upon stimulation (Figure 3A), whereas only a small fraction of the cellular proteome significantly changed in abundance (Figure 3B).

### The Immunopeptidome Correlates With Protein Half-Life but Not Cellular Protein Abundance

Previous studies suggested that HLA molecules are more likely to present peptides derived from proteins with high turnover rate (13, 15). To examine this property in our data, we extracted the published half-lives of the presented proteins that we identified from the database of Larance et al. (55) which includes a measure of degradation, or protein turnover ratio, for the proteome. Indeed, we found that the mean protein turnover ratio for the parent proteins of the immunopeptidome is lower than that of the cellular proteome (Figure 3C; Mann-Whitney ^*^p = 0.0182). Likewise, using the GeneCards Suite Human Integrated Protein Expression Database (HIPED) (56), we found that cellular proteins, which are presented, had lower abundance (on average) than the proteins that are not presented (Figure 3D; Mann-Whitney ^****^P < 0.0001). Finally, as was shown previously (15), the number of unique peptides presented from a given protein modestly correlates with the abundance of the protein in the cell (Supplementary Figure 6A) and this correlation was not influenced by cytokine stimulation (Supplementary Figure 6B). This held true even when the number of peptides was normalized by the length of the protein (Supplementary Figures 6C,D). However, when we compared the subset of proteins that increased in abundance due to the TNFα and IFNγ stimulation to proteins that did not, we found no correlation (Figure 3E), suggesting that the increase in protein abundance is not driving peptides for presentation on HLA. However, there was a small fraction of proteins (*n* = 110) which increased in abundance after stimulation by 2-fold or more and is enriched in responders to IFNγ and general immune system processes (Supplementary Figure 4B). In this fraction of cellular proteins, the correlation with presented protein abundance increases to *r* = 0.33 (Supplementary Figure 6E). To rule out that the lack of correlation is due to the different dynamic ranges of the immunopeptidome and cellular proteome, we ranked the fold changes of all the proteins in both datasets. Still, the analysis showed no correlation in the changes observed in the immunopeptidome and cellular proteome (Supplementary Figure 6F). We note that this observation may be due to the specific time points at which we examined the data, which may not capture a biological correlation between these two processes. Nevertheless, our results indicate that the half-life of proteins, rather than their abundance, may be an important determinant in the regulation of the immunopeptidome.

### The Upregulation of HLA-B Changes the Presented Peptidome

**Figure 4:**
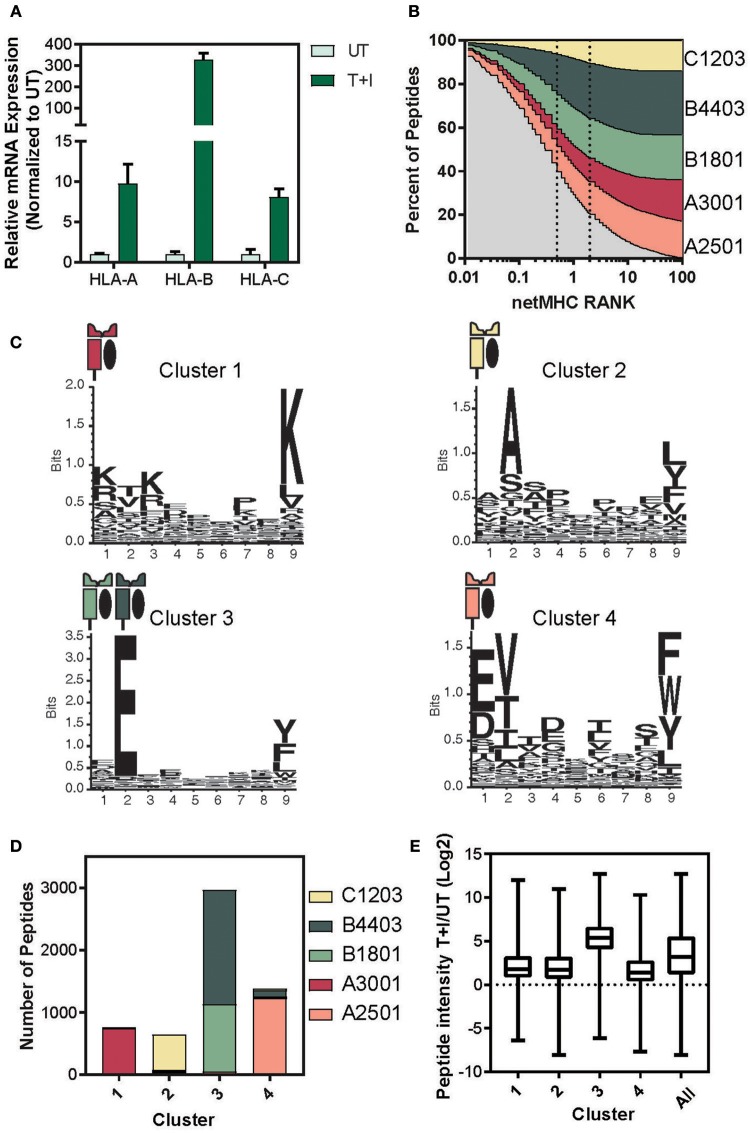
Determining the haplotype composition of A549 cells. **(A)** Relative levels of mRNA expression of HLA- A, B, and C with TNFα and IFNγ stimulation (T+I) or unstimulated (UT). GAPDH was used as an internal normalizing control and each gene was normalized to the UT sample. **(B)** NetMHC 4.0 was used to predict which of the identified presented peptides would bind to one of the HLA haplotypes found in A549 cells. The percentage of peptides identified which are predicted to bind to each haplotype is plotted against the threshold of binding rank (NetMHC RANK). The typical strong (rank = 0.5) and weak (rank = 2) binding threshold are indicated on the graph. **(C)** Gibbs clustering was performed on the identified peptides with four clusters. The motif of the peptides in each of the four clusters is displayed. **(D)** The number of peptides in each cluster predicted to bind to one of the A549 haplotypes. **(E)** The log2 transformed ratio of presented peptide intensity between cells stimulated with TNFα + IFNγ (T+I) and unstimulated (UT) for the peptides in each cluster (line at median, box for the second and third quartile, lines from min to max).

It has previously been reported that HLA-B expression increases more strongly than HLA-A and -C upon IFNγ signaling (38–42). Based on this we examined the relative expression levels of the three HLA loci. We found consensus sites between the two haplotypes of each locus that had at least 3 mismatches with the other two loci (see materials and methods section for primer design). Therefore, each primer set amplifies the A, B, or C transcript of HLA (Supplementary Figure 7A). Using these primers, we performed quantitative real-time polymerase chain reaction (qPCR) and found that HLA-A, B, and C significantly increased in A549 cells upon stimulation for 24 h (Figure 4A). Furthermore, the HLA-B transcript level increased 30-fold more than the increase in HLA-A, as was described in Komov et al. (42).

Given that there is a significant change in the relative levels of the different HLA loci upon stimulation, we predicted this might explain much of the change in the resulting immunopeptidome. Thus, we matched each peptide identified in the immunopeptidome to the HLA haplotype it would be predicted to bind to, using NetMHC (57, 58). Almost 80% of the peptides identified were predicted to bind an MHC with a binding rank of 2 or lower, suggesting a high binding affinity (Figure 4B). Examining the binding constraints of the allomorphs of the A549 cell line, we found that both HLA-B4403 and -B1801 bind mainly to peptides with a glutamic acid at the second position (Supplementary Figure 7B).

To cluster peptides based on their biochemical properties, we utilized the Gibbs clustering method and separated the immunopeptidome into four distinct clusters based on their sequence motifs (Supplementary Figure 8A; Supplementary Table 2). Indeed, we found that the peptides in each of the other clusters predominantly bind to a single haplotype and that Cluster 3 reflected a known motif of HLA-B1801 and -B4403 (Figure 4C). Indeed, when we examined the percentage of each cluster predicted to bind to each of the A549 HLA haplotypes, we found that over 90% of the peptides in cluster 3 were predicted to bind to the HLA-B haplotypes (Figure 4D and Supplementary Figure 8B). In accordance with these findings, the peptides in cluster 3 exhibited a greater increase in intensity as a result of the cytokine stimulation (Figure 4E). We did not observe such changes neither in measured abundance of the cellular parent proteins (Supplementary Figure 8C) nor in the HIPED abundance across the four clusters (Supplementary Figure 8D). This indicates that the increase in the abundance of peptides predicted to bind to HLA-B is due to the increase in the expression of HLA-B and not due to a change in the abundance of the cellular proteins.

### Changes in the Cleavage and Binding of HLA Peptides Following Pro-Inflammatory Cytokine Stimulation Alters the Immunopeptidome Landscape

Upon establishing that more HLA-B bound peptides are presented following stimulation, we next assessed the impact this has on the immunopeptidome landscape. Indeed, cluster 3 showed the greatest percentage of peptides, which were unique to a given protein (Supplementary Figure 9A). We then calculated the mean number of peptides presented per protein (sampling density). Across the four Gibbs clusters, only cluster 3 increased significantly in mean sampling density upon stimulation. (Supplementary Figure 9B; ^**^p = 0.0018, ^****^p < 0.0001). Importantly, this was in contrast to the representation of peptides from cluster 3 prior to stimulation, which had the lowest peptide density as compared to the other clusters (Supplementary Figure 9B). Nevertheless, this was not a result of changes in cellular protein abundance. We found that the correlation between the number of peptides presented from a protein and the protein abundance in the cell remained constant across all clusters and treatments (Supplementary Figures 9C,D).

**Figure 5:**
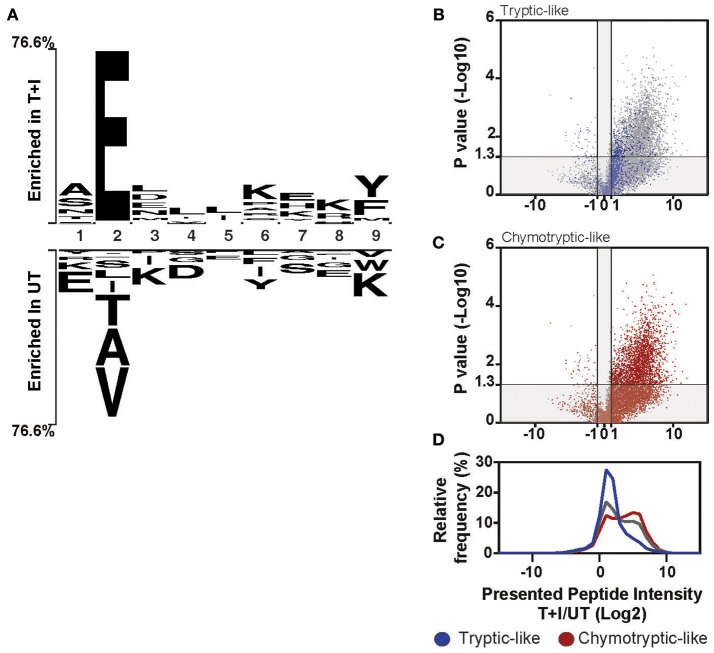
Induction of HLA-B changes the overall motif of presented peptides. **(A)** The motif of the top 25% (Enriched in T+I) of 9-mer peptides based on the ranking in Figure S9E is compared to the bottom 25% (Enriched in UT). The residues that are enriched at each position are displayed above or below the axis **(B–D)** Peptides were classified as tryptic-like (K, R c-terminal residues) or chymotryptic like (A,F,I,L,M,V,Y c-terminal residues). The fold change in intensity for each peptide between TNFα and IFNγ stimulated (T+I) and unstimulated cells (UT; Log2 transformed) was plotted against the *p*-value for the difference between the two treatment groups (two-tailed *t*-test, –Log10 transformed). The peptides, which are tryptic-like (**B**, blue dots) or chymotryptic-like (**C**, red-dots), are overlaid on the background of all peptides identified along with the histogram of intensity fold changes for these three groups of peptides (**D**; tryptic-like, chymotryptic-like and other).

These changes in the composition of the immunopeptidome also affected the overall sequence motif. When we looked at each peptide ranked by the amount of increase in abundance following stimulation, we found that over 80% of the peptides in the top quartile contained a glutamic acid at the second position of the peptide (Supplementary Figures 9E–G). Further, when we examined the sequence of the 9-mer peptides in the top and bottom quartile, we found a motif very much resembling the HLA-B binding motif was enriched in the stimulated immunopeptidome, while a motif resembling the binding motif of HLA-A2501 was enriched in the unstimulated pool (Figure 5A). Thus, pro-inflammatory cytokine stimulation changed the repertoire of the presented peptides, skewing it to peptides with glutamic acid at the second position, also corresponding to the binding motif of HLA-B.

Notably, the cleavage motif of the presented peptides shifted in accordance with the increased chymotryptic-like activity of the immunoproteasome as observed by analysis of the carboxyl-terminal (c-terminus) residue of the presented peptides. When comparing the motif of the stimulated and unstimulated peptidome, the most represented residue at the c-terminus of the peptide is tyrosine (Y) and lysine (K), respectively (Figure 5A). The tryptic-like activity of the proteasome is responsible for cleavages after lysine, while the chymotryptic-like activity cleaves after tyrosine. Indeed, when we classified all the peptides with cleavage patterns resulting from the chymotryptic-like or tryptic-like cleavage activity of the proteasome, chymotryptic-like peptides increased more in intensity following stimulation as compared to tryptic-like peptides (Figures 5B–D). This correlates with the additional chymotryptic-like cleavage activity of the immunoproteasome as discussed previously (11, 36, 59).

**Figure 6:**
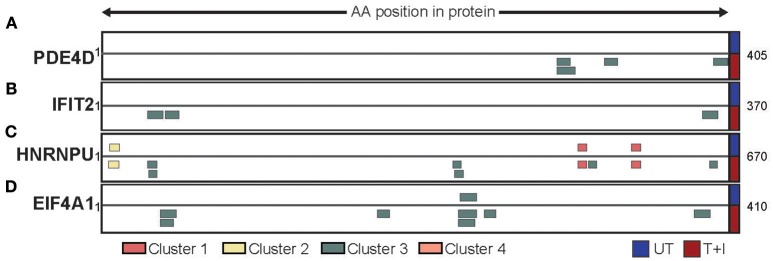
Different protein repertoires presented following stimulation. The presented peptides from **(A)** PDE4D, **(B)** IFIT2, **(C)** HNRNPU, and **(D)** EIF4A1 are shown aligned to their position on the protein sequence. Only the portion of the protein which had peptides identified is shown (start and end position are marked) and peptides are colored by the cluster which they were assigned to. The peptides identified in unstimulated cells (UT) are presented above the protein while the peptides identified in stimulated cells (T+I) are below.

Based on our analyses we have identified three ways by which the stimulation affected the presented peptidome. First, we found that, of the proteins containing peptides presented on HLA following stimulation, 22% (845 proteins) were only presented on HLA-B. Two examples of such proteins are PDE4D (cAMP-specific 3′,5′-cyclic phosphodiesterase 4D; Figure 6A) and IFIT2 (Interferon Induced Protein with Tetratricopeptide Repeats 2; Figure 6B). The former is a protein which is not expected to change in abundance in the cell following stimulation, while the second is known to be induced by IFNγ. Second, we found proteins which contained peptides that are presented both in unstimulated cells and following stimulation. However, the availability of new HLA complexes with different binding constrains allows for new areas of the protein (i.e., peptides) to be presented. This is the case with HNRNPU (Heterogeneous nuclear ribonucleoprotein U) which contains cluster 1 and 2 peptides that were identified in both unstimulated and stimulated conditions, as well as cluster 3 peptides that were only identified following stimulation (Figure 6C). Finally, there are proteins such as EIF4A1 (Eukaryotic initiation factor 4A–I) that have a peptides presented from them in low quantity, but stimulation increases the number of peptides presented from the same region of the protein (Figure 6D). Through these avenues, cytokine stimulation induces a change in the expression of HLA-B, in turn increases and changes the repertoire of peptides detected.

### HLA-B Expression Correlates With Signature of Tumor Inflammation and T Cell Infiltration in TCGA Lung Cancer Cohort

**Figure 7:**
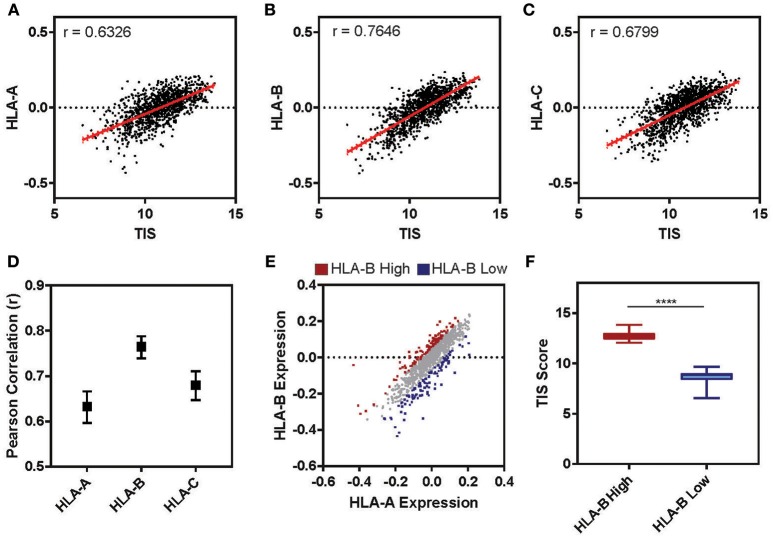
Tumor Inflammation Signature correlates with relative levels of HLA-B across patients. **(A–C)** The relative expression of HLA-A **(A)**, HLA-B **(B)**, or HLA-C **(C)** (normalized to the mean expression for that gene in the cohort and log2 transformed) is plotted against the tumor inflammation signature (TIS). The Pearson correlation (r) for these two parameters across the full TCGA lung cohort (*n* = 1,128) is displayed on the graph. **(D)** The Pearson correlation for the analysis presented in (A–C). Upper and lower limits on the correlation are indicated by bars. **(E)** The relative expression of HLA-A is plotted against HLA-B for the cohort (*n* = 1,128). Each sample was ranked by the difference between HLA-B and HLA-A expression and the 10% at the top (HLA-B high) and bottom (HLA-B low) of the ranking are marked in red and blue, respectively. **(F)** The mean TIS score for the two subsets defined in E are displayed. The HLA-B high subset has a significantly higher average TIS score (Mann Whitney, two tailed *T*-test, ^****^p < 0.0001; whiskers indicate min and max).

The tumor microenvironment (TME) is largely shaped by immune cell infiltration into the tumor and cancer-related inflammation as reviewed in (60, 61). In many cases, “cold” tumors for which low levels of immune infiltration is observed are associated with non-responsiveness to therapy and worse prognosis (62–64). To test whether the expression level of HLA-B is correlated with the inflammatory state of a tumor in clinical samples, we examined the cancer genome atlas (TCGA) lung cohort (*n* = 1,128). For each patient we calculated a tumor inflammation signature (TIS) value based on the linear weighted sum of eighteen genes (Supplementary Table 3) involved in interferon activity, T cell and NK cell abundance, T cell exhaustion and antigen presenting cell abundance (65). While the TIS was positively correlated with the relative expression of all three HLA I genes (Figures 7A–C), HLA-B was correlated with the TIS score to a higher degree than either HLA-A or -C (Figure 7D; HLA-B *r* = 0.76, HLA-A *r* = 0.63, HLA-C *r* = 0.68). Ranking tumors based on their relative expression of HLA-B compared to HLA-A allowed us to define two subclasses of tumors expressing high HLA-B (denoted “HLA-B high”) and low HLA-B (“HLA-B low”) (Figure 7E). Indeed, the HLA-B high tumors had a significantly higher TIS score on average as compared to the HLA-B low cohort (Figure 7F; Mann-Whitney ^****^p < 0.0001). This suggests that tumor samples with a higher relative expression of HLA-B also had increased expression of inflammation and immune infiltration markers.

## Discussion

Inflammation is a key hallmark of the TME (66) which is mediated by a mixture of cytokines that are secreted into the tumor environment both by tumor and immune cells. TNFα and IFNγ, for example, were shown to have both promoting and inhibitory effects on tumor growth (67–69). Aside from the role of IFNγ in immunoediting, thereby preventing primary tumor formation (70), defects in both IFN (71) and TNF (72) signaling have been associated with increased cancer risk. Furthermore, TNFα and IFNγ in combination induce tumor cell senesce (73) mediated by CD4^+^ T cells (Th1) (73, 74). Importantly, IFNγ and TNFα were previously shown to function synergistically to modulate the expression levels of the antigen processing and presentation machinery (43–47). This pro-inflammatory cytokine stimulation has been shown to induce expression of the immunoproteasome and modulate the relative expression of the different HLA genes, particularly HLA-B (38–41, 75–78). Recent studies examined the effects of IFNγ stimulation on the generation of specific antigenic peptides (37, 79–81) or the general immunopeptide landscape (36, 42), suggesting that IFNγ induced proteasome-mediated changes to the presented peptidome. However, the HLA-B mediated effects on the immunopeptidome were first described in the work by Komov et al. (42). They found that there are more HLA-B presented peptides following cytokine stimulation and that the proteasome-cleaved peptide supply of ligands for loading were not the limiting factor in presentation. Rather, they could show that the changes they observed to the immunopeptidome of MCF7 cells were driven by a limited availability of HLA complexes. However, this analysis was done in response to IFNγ and IFNβ stimulation which served to increase HLA expression.

In this work, we wished to examine the changes in the immunopeptidome landscape under conditions mimicking the pro-inflammatory signals that are likely to exist in the TME. To that end, we stimulated lung cancer cells, A549, with TNFα and IFNγ and analyzed the changes in both the cellular proteome and HLA presentation. Our analysis revealed that HLA-B becomes the dominant haplotype of these cells after stimulation, whereas in unstimulated A549 cells it is hardly expressed. This, in turn, causes the sequence motif of the presented peptides to change dramatically, in accordance with the binding constrains of the different HLA haplotypes. Likewise, a shift in cleavage properties of the presented peptides is observed, with an increase in chymotryptic-like peptides. In addition, the protein repertoire represented by the immunopeptidome is both altered and diversified following TNFα and IFNγ stimulation. Importantly, we show that these changes are not driven by changes in the relative abundances of the cellular proteins but rather as an outcome of the change in the antigen presentation machinery. This further supports the finding that peptides are not the limiting factor in determining the immunopeptide repertoire (42) and additional factors operate to select and actively regulate the peptides that are presented on cellular HLA molecules.

Taken together, our results support the notion that a cellular mechanism for induction of HLA-B upon inflammatory stimulation exists (36, 38–42), and suggest that such modulation of the immunopeptidome may also be important in clinical settings. In contrast to previously reported determinants of the immunopeptidome under steady state conditions, this study presents a mechanism whereby the peptidome can be modulated in both proteome- dependent and independent manners. These results emphasize the importance of studying the immunopeptidome in a context as close as possible to the physiological conditions in order to improve the identification of presented peptides. Indeed, in recent years, much effort has been put into identifying peptides specifically presented by cancerous cells, in an effort to elicit anti-tumor immunity. Many of the methods employed to identify these tumor-specific peptides, or neo-antigens, rely on expanding tumor biopsies in culture, thereby removing the cells from their physiological contexts. TNFα and IFNγ are often expressed in the tumors' microenvironment and these signals may be altered when tumor cells are passaged in culture. Our results suggest that the state of inflammation in the microenvironment of a tumor affects the relative expression of the three different HLA genes. Our TCGA analysis strengthens the relevance of our findings to human lung cancer and its TME. This suggests that inter-patient variation in the TME will, in turn, modulate the patient's immunopeptidome repertoire and may affect tumor host-interactions. Further study is needed to ascertain the relative antigenicity of the differentially presented peptides, as well as their ability to elicit an immune response via tumor infiltrating lymphocytes. Nevertheless, we show that the full extent of cis and trans-regulation of the HLA immunopeptidome depends on both cell autonomous processes as well as exogenous. Revealing the principles underlying selection and presentation of peptides, across various cellular conditions may harness novel opportunities for shaping and modulating antigenicity in cancer therapy.

## Materials and Methods

### Cell Culture and Treatments

A549 cells were grown in DMEM supplemented with 10% fetal bovine serum, 1% Penicillin/streptomycin and L-glutamine (2 mM) (Biological industries) at 37°C with 5% CO_2_. Cells were treated with 400 U^*^mL-1 TNFα and/or 200 U^*^mL-1 IFNγ (peprotech) for the indicated amount of time.

### Antibodies

For flow cytometry, HLA complexes were stained with PE conjugated W6/32 pan-HLA (Biolegend, BLG-311405). For Western Blots the following antibodies were used: β5 (Enzo | bml-pw8895 | lot # 04181631 | 1:1000), β5i (Abcam | ab180606 | lot # F1716 | 1:1000), β1i (Sigma Aldrich | SAB4200270 | lot # GR3191928-2 | 1:1000), beta actin (ThermoFisher Scientific|MA5-15739 | lot # 32253598 | 1:1000), Goat anti-Mouse IgG-HRP (Jackson | JIR 115-035), Goat anti-Rabbit IgG-HRP (Jackson | JIR 111-035).

### Western Blot Analysis

About 2.5 × 10^6^ A549 cells per condition were collected and flash frozen. Cells were then suspended in radio-immunoprecipitation assay buffer (150 mM NaCl, 1.0% NP-40, 0.5% sodium deoxycholate, 0.1% SDS, 50 mM Tris, pH 8.0), incubated on ice for 30 min and centrifuged at 21,130 rcf for 30 min. Protein concentrations were measured using the Coomassie Plus (Bradford) Assay Kit (Pierce, ThermoFisher Scientific). For each condition, 20 μg of cellular lysate was incubated at 95°C for 5 min, then separated by SDS-PAGE and transferred to nitrocellulose membranes (iBlot Transfer Stack, ThermoFisher Scientific) which were incubated with the indicated primary antibodies, followed by the appropriate secondary antibody conjugated to horseradish peroxidase. Enhanced chemi-luminescence was acquired in a Molecular Imager Gel Doc XR System (BioRad).

### RT-PCR Analysis

RNA was extracted and purified from cells using the Direct-Zol RNA kit (Zymo Research, R2052). mRNA levels were ascertained by real time quantitative PCR using SYBR Green (Kapa Biosystems) and primers as outlined in Table 1.

**Table 1:** Quantitative real-time PCR primer sequences.

**Gene**	**Forward**	**Reverse**
HLA-A	TGGAGCTGTGATCACTGGAG	GGGCACTGTCACTGCTTG
HLA-B	AGACACAGATCTCCAAGACCAACA	CGTCGCAGCCGTACATCCT
HLA-C	CTGTCCTAGCTGTCCTAGGAGCT	CCTGGGCACTGTTGCTGG
GAPDH	CAACGGATTTGGTCGTATTG	GATGACAAGCTTCCCGTTCT

### Proteasome Cleavage Reporter Assay

A549 cells, stimulated with TNFα and IFNγ for the indicated times, were collected and flash frozen. Frozen cells were re-suspended in lysis buffer (25 mM sucrose, 50 mM TRIS pH 7.4, 5 mM MgCl_2_, 0.5 mM EDTA, 2 mM ATP, 1 mM DTT). Lysates were passed 10 times through a 27 G needle, incubated on ice for 15 min and the centrifuged at 21130rcf for 10 min. Protein concentrations were measured using the Coomassie Plus (Bradford) Assay Kit (Pierce, ThermoFisher Scientific). 20 μg of cellular lysate was incubated with 0.1 mM suc-LLVY-AMC or ac-PAL-AMC (Biotest) as per protocol and fluorescence levels were measured over time using a BioTek Synergy H1 plate reader (Ex: 360 nm, Em: 460 nm). The background protease activity was determined for each condition from an identically prepared sample with the addition of Mg132 proteasome inhibitor (0.04 mM; Calbiochem). Each time point measurement was performed in three independent biological replicates.

### Cell Viability Assay

To measure cells' ATP content, 7500 A549 cells were seeded per well in a 384-well plate. After 36 h, including stimulation with TNFα and IFNγ for the indicated time, a half volume of the CellTiter-Glo reagent (Promega) was added to each well following (82). The reagent was incubated with the cells for 10 min per the manufacturer's protocol and then luminescence levels were measured over time using BioTek Synergy H1 plate reader.

### Immunoaffinity Purification of the HLA Complexes

Membranal HLA (mHLA) class I molecules were purified similarly to Hunt et al. (83) with modifications as described in Milner et al. (84) with minor modifications, from about 5 × 10^8^ cells. The cells were lysed with 0.25% sodium deoxycholate, 0.2 mM iodoacetamide, 1 mM EDTA, 1:200 Protease Inhibitors Cocktail (Sigma Aldrich, St. Louis, MO), 1 mM PMSF and 1% octyl-β-D glucopyranoside (Sigma Aldrich) in PBS at 4°C for 1 h. Cell extracts were cleared by centrifugation for 45 min, at 48,000 g and at 4°C. The HLA class I molecules were immunoaffinity purified using the W6/32 mAb bound to protein A-resin beads (Genscript, Piscataway, NJ) as in Barnea et al. (85). The HLA molecules with their bound peptides were eluted from the affinity column with five column volumes of 1% TFA. The eluted HLA class I proteins and the released peptides were loaded on disposable C_18_ columns (Harvard Apparatus, Holliston, MA) and the peptides fraction was recovered with 30% acetonitrile in 0.1% TFA, as in Milner et al. (84). The peptides were dried using vacuum centrifugation, reconstituted with 100 μl of 0.1% TFA, reloaded on C_18_ StageTips, prepared as in Rappsilber et al. (86), eluted with 80% acetonitrile, dried and reconstituted with 0.1% formic acid for the LC-MS/MS analysis.

### Identification of HLA Peptides

The LC-MS/MS analyses of the HLA and the tryptic peptides were performed with a Q-Exactive-Plus mass spectrometer fitted with either with Easy nLC 1000 capillary HPLC (Thermo-Fisher Scientific) or with Ultimate 3000 RSLC nano-capillary UHPLC (Thermo-Fisher Scientific). The reversed phase chromatographies were performed with home-made 30 cm long, 75 μm inner diameter, packed with 3.5 μm silica ReproSil-Pur C18-AQ resin (Dr. Maisch GmbH, Ammerbuch-Entringen, Germany), as in Ishihama et al. (87). The HLA peptides were eluted using a linear gradient of 5–28% of acetonitrile in 0.1% formic acid, at a flow rate of 0.15 μl/min for 2 h. Data was acquired using a data-dependent “top 10” method, fragmenting the peptides by higher-energy collisional dissociation (HCD). Full scan MS spectra were acquired at a resolution of 70,000 at 200 m/z with a target value of 3 × 10^6^ ions. Fragmented masses were accumulated to AGC (automatic gain control) target value of 10^5^ with a maximum injection time of 100 ms. No fragmentation was attempted for HLA peptides with unassigned precursor charge states. The peptide match option was set to Preferred. The normalized collision energy was set to 25% and MS/MS resolution was 17,500 at 200 m/z. Fragmented m/z values were dynamically excluded from further selection for 20 s.

### Cellular Lysates Preparation

Tryptic Digest: Pellets were suspended in 5% SDS, pH 7.5, then heated for 3 min at 95°C. Lysates were then sonicated for 6 cycles (Diagnode). Protein concentration was measured using a BCA assay. Proteins were reduced using 5 mM dithiothreitol and alkylated using iodoacetamide. Samples were then loaded onto an S-trap column (Protifi, USA) and subjected to in-solution tryptic digestion according to the manufacturer's protocol. The samples were vacuum dried and stored in −80°C until analysis.

### Identification of Cellular Proteins

Tryptic Digests: ULC/MS grade solvents were used for all chromatographic steps. Each sample was loaded using split-less nano-Ultra Performance Liquid Chromatography (10 kpsi nanoAcquity; Waters, Milford, MA, USA). The mobile phase was: A) H2O + 0.1% formic acid and B) acetonitrile + 0.1% formic acid. Desalting of the samples was performed online using a reversed-phase Symmetry C18 trapping column (180 μm internal diameter, 20 mm length, 5 μm particle size; Waters). The peptides were then separated using a HSS T3 nano-column (75 μm internal diameter, 250 mm length, 1.8 μm particle size; Waters) at 0.35 μL/min. Peptides were eluted from the column into the mass spectrometer using the following gradient: 4–30%B in 163 min, 30–90%B in 5 min, maintained at 90% for 5 min and then back to initial conditions. The nanoUPLC was coupled online through a nanoESI emitter (10 μm tip; New Objective; Woburn, MA, USA) to a quadrupole orbitrap mass spectrometer (Q Exactive Plus, Thermo Scientific) using a FlexIon nanospray apparatus (Thermo). Data was acquired in data dependent acquisition (DDA) mode, using a Top10 method. MS1 resolution was set to 70,000 (at 400 m/z), mass range of 300–1,650m/z, AGC of 3e6 and maximum injection time was set to 50 ms. MS2 resolution was set to 17,500, quadrupole isolation 1.7 m/z, AGC of 1e5, dynamic exclusion of 60 s and maximum injection time of 60 ms.

### Mass Spectrometry Data Analysis

Peptides were identified and quantified by the MaxQuant software [version 1.6.0.16 (51)] with the following parameters: unspecific enzyme, minimum peptide length for unspecific search of 8, maximum peptide length for unspecific search of 25, and match between runs enabled. Methionine oxidation and N-acetylation were accepted as variable modifications. A false discovery rate (FDR) of 1% was applied for peptide identification. For the analysis of tryptic digests, the MaxQuant default parameters were set. Masses were searched against the human proteome database from the Uniprot/Swiss-Prot (last update on 2.2018). The MS proteomics data of the cellular proteome and immunopeptidome have been deposited to the ProteomeXchange Consortium via the PRIDE (88) partner repository with the dataset identifiers PXD009935 and PXD009936.

### Label-Free Quantitation and Bioinformatics Analysis

Using Python 3.6, peptides identified though MaxQuant (50) were initially filtered to remove reverse sequences and known MS contaminants. We identified 7,524 unique peptides across both treatments with an average of 3,508 and 6,816 peptides in the untreated and stimulated triplicate samples, respectively. To reduce ambiguity, we allowed peptides that had at least two valid LFQ intensities out of three independent biological replicates in at least one treatment group, and included razor peptides, which belong to a unique MaxQuant “protein group.” Following this filtering step we were left with an average of 3,444 peptides in the untreated samples and 6,582 peptides in the samples. Finally, missing peptide intensity values were imputed to randomly chosen values from a normal distribution. These intensity values are then used as a relative measure of peptide abundance. Following the generation of the lists of identified peptides, the third triplicate of untreated cells (UT-3) was excluded from the analysis, as fewer than half the number of peptides were identified in UT-3 as compared to UT-1 and −2. In addition, the sample was poorly correlated with the other samples in the triplicate. Protein abundances were inferred from peptide intensities using MaxQuant. For graphical representation, intensities were log transformed, and zero intensity were imputed to a random value chosen from a normal distribution of 0.3 the standard deviation and downshifted 1.8 standard deviations (89). Peptide and protein intensities are reported as the median intensity across the triplicate (or duplicate for UT) unless noted otherwise.

The identified HLA peptides were subject to Gibbs clustering using the default parameters for MHC I peptides (90, 91). The haplotype of A549 cells was previously reported in (92). Identified peptides were assigned to their best fit MHC using NetMHC 4.0 for the HLA haplotypes, which are present in A549 and for which information is available in NetMHC (57, 58). The sequence logo of peptides bound by each A549 haplotype were determined through Gibbs clustering of all of the peptides annotated as binding to that haplotype in the IEDB ]Immune Epitope Database and Analysis Resource; (93)]. All motifs (with the exception of Figure 5A) were generated by Seq2Logo 2 using default parameters except for the logo type, which was set to Shannon (94). The comparison motif in Figure 5A was generated using Two Sample logo (95). Default parameter were used with the exception of bonferroni correction which was enabled.

Standard scores were calculated as follows:

$$\begin{array}{ccc} z & = & \frac{{\bar{x}}_{stimulated}-{\bar{x}}_{unstimulated}}{\sqrt{\frac{\sigma_{stimulated}^{2}}{n_{stimualted}}+\frac{\sigma_{unstimulated}^{2}}{n_{unstimualted}}}} \end{array}$$

Whole protein turnover rates were those published by Larance et al. (55). They are presented as log2 transformed ratios between the abundance following treatment with cycloheximide (CHX) and DMSO. Because CHX inhibits protein synthesis, general protein abundance decreases resulting in negative fold changes. Protein abundance data was mined from the GeneCards Suite Human Integrated Protein Expression Database (HIPED) (56), unifying protein abundance data from 4 proteomics sources, ProteomicsDB, MOPED, PaxDb, and MaxQB. We utilized HIPED's integrated proteomes of 69 normal human anatomical entities (tissues, *in-vivo* cells and body fluids). The mined protein abundance PPM (parts per million) values were normalized by log10 transformation. All statistical analyses were performed using either GraphPad Prism or MATLAB 2016a. Pathway enrichment was done using Panther 13.1 [Protein Analysis Through Evolutionary Relationships (54)] using the GO-slim biological processes annotation collection. Protein schematics were made using the start and end position of the presented peptides aligned to the protein sequence. Only peptides with a log10 transformed intensity >6 are displayed.

### TCGA Data Analysis

TCGA data was mined using the xenaPython package in Python 3.6. The results shown in this analysis are in whole or part based upon data generated by the TCGA Research Network: http://cancergenome.nih.gov/. The full lung carcinoma cohort (both lung adenocarcinoma [LUAD] and lung squamous cell carcinoma [LUSC] designations) was used for this study. One sample (TCGA-63-A5MY-01) was removed as an outlier because the expression of HLA-B was more than 3 interquartile ranges below quartile Q1 leaving a total of *n* = 1,128 samples for the study. The Tumor Inflammation Signature, or TIS was calculated as the weighted linear sum of the expression levels of 18 genes (Supplementary Table 3) as developed by Ayer et al. and Danaher et al. (65, 96). Expression levels of the three HLA genes were normalized to the mean expression for that gene in the cohort and log2 transformed.

## Author Contributions

AJ and YM conceived and planned the experiments. AJ, EB, MK, and HW-L carried out experimental work. AJ performed the data analysis. YL, AA, and YM supervised the findings of the work. AJ and YM wrote the manuscript and all authors discussed the results and contributed to the final manuscript.

### Conflict of Interest Statement

The authors declare that the research was conducted in the absence of any commercial or financial relationships that could be construed as a potential conflict of interest.

## Supplementary Material

Supplementary Figure 1 Flow Cytometry analysis measuring the levels of HLA on the surface of A549 cells after treatment with TNFα and IFNγ for the indicated times. **(A)** Histograms of the fluorescence intensity corresponding to the surface expression of HLA I using PE-W6/32 (PE: HLA) and **(B)** the mean fluorescence intensity (MFI) are displayed. **(C)** Whole cell lysate from cells stimulated with TNFα and IFNγ for the indicated times was separated on SDS-PAGE and analyzed by western blot probed with the indicated antibodies. Actin is displayed as a loading control. **(D)** Cells stimulated with TNFα and IFNγ for the indicated times were incubated with CellTiter-Glo reagent and the measured luminescence levels were plotted (y-axis, ATP levels). Each dot represents a biological repeat of the experiment. There was no significant difference between 0 h of stimulation and 24 h (*p* = 0.1314).

Supplementary Figure 2 **(A)** Scatter plot of the HLA presented peptide abundances (Log10) between each pairwise combination of samples. Along the diagonal of the graph, the histogram of abundance values (before imputation) for each of the samples.

Supplementary Figure 3 **(A,B)** The median intensity of each peptide across three (x-axis) or two (y-axis) replicates from the unstimulated samples (UT; **A**) or TNFα and IFNγ (T+I) stimulated samples **(B)**. A red dashed line is plotted at X = Y. **(C)** The mean intensity of the peptide population from two or three replicates of the unstimulated samples (UT) or TNFα and IFNγ (T+I) stimulated cells. The bars in red represent the analysis conditions chosen for the manuscript.

Supplementary Figure 4 **(A)** Volcano plot of the proteins identified in unstimulated cells (UT) or cells stimulated with TNFα + IFNγ (T+I). Ratios are Log2 normalized and *P*-values are Benjamini Hochberg FDR corrected and –Log10 transformed. Proteins enriched in T+I are marked in red, enriched in UT are marked in blue and those which contain a presented peptide are marked in a black outline. **(B)** Significantly enriched or underrepresented biological processes in the protein subset increased in abundance after stimulation with TNFα + IFNγ (2-fold increase or greater in abundance). All the processes passed an FDR cut-off of 0.05 and the log2 transformed normalized enrichment score is presented for each process.

Supplementary Figure 5 **(A)** A Venn diagram showing the number and percentage of proteins identified only in the cellular proteome, only in the presented proteome or shared by both.

Supplementary Figure 6 **(A,B)** For either unstimulated (UT) cells **(A)** or cells stimulated with TNFα + IFNγ (T+I, **B**) the abundance of each protein in the cell was plotted against the number of unique peptides presented from that protein in stimulated cells. The red line indicates a moving average with a window of 50. Pearson correlation (r) is displayed for each plot. **(C,D)** For either unstimulated (UT) cells **(C)** or cells stimulated with TNFα + IFNγ (T+I, **D**) the abundance of each protein in the cell was plotted against the number of unique peptides presented from that protein and normalized to the protein length. The red line indicates a moving average with a window of 50. Pearson correlation (r) is displayed for each plot. **(E)** The ratio of cellular protein abundance between stimulation with TNFα + IFNγ (T+I) and unstimulated (UT) is plotted against the ratio in intensity for the presentation of each protein (Log2 transformed ratios). Proteins which increased by 2-fold or more in cellular abundance and increased in presented abundance are marked in red (*n* = 110). The Pearson correlation (r) for this subset is displayed on the graph (*r* = 0.325). **(F)** The ratios in Figure 3E are ranked and the ranks are plotted against one another (Log2 transformed ratios, Pearson correlation (r) is displayed on the graph, *n* = 1239).

Supplementary Figure 7 **(A)** Schematic overlay of the HLA transcripts with the differential regions used to design primers. **(B)** The motifs of peptides known to bind to each of the five A549 haplotypes.

Supplementary Figure 8 **(A)** The Kullbakck-Leibler distance for Gibbs clustering performed with between 1 and 5 different clusters. **(B)** The percent of peptides in each cluster predicted to bind to one of the A549 haplotypes. **(C)** The log2 transformed ratio of protein abundance in the cell between stimulation with TNFα + IFNγ (T+I) and unstimulated (UT) for the parent proteins of the peptides in each cluster (line at median, box for the second and third quartile, lines from min to max) **(D)** Protein abundance was inferred from the GeneCards Suite Human Integrated Protein Expression Database (HIPED) (56) and applied to the subset of proteins in each Gibbs cluster.

Supplementary Figure 9 **(A)** The percentage of the peptides contained in each of the 4 clusters which are peptides unique to a given protein. **(B)** The average number of peptides presented from a given protein (HLA sampling density) for each of the four clusters across both unstimulated (UT) or stimulated (T+I) conditions (^**^*p* = 0.0018, ^****^*p* < 0.0001; error bars indicate 95% confidence intervals on the mean). **(C)** The rolling average of the HLA sampling density (y axis) ranked by the abundance of the protein in the cellular lysate (x axis; rolling window = 50). Peptides were subdivided by both treatment condition and cluster. **(D)** The Pearson correlation between the HLA sampling density of a given protein and the abundance of that protein in the cell for each cluster and treatment combination (error bars indicate 95% confidence intervals on the correlation). **(E)** Each HLA presented peptide was ranked based on the change in intensity between TNFα and IFNγ stimulated and unstimulated cells (X axis is shared with G). Each protein was assigned a standard score (Z score) based on the significance of the deviation. **(F)** The amino acid at the second position of each peptide (X-axis is shared with G) is marked with a blue (A, V), red **(E)** or black (remaining amino acids). **(G)** The percentage of peptides which have a glutamic acid at their second position was calculated using a rolling window of 200 peptides across peptides ranked based on the change in intensity between TNFα and IFNγ stimulated and unstimulated cells.

Supplementary Table 1 The proteins identified in WCE in untreated cells and following cytokine stimulation along with fold change in abundance and corrected *P*-value.

Supplementary Table 2 The peptides identified in the immunopeptidome of untreated and stimulated cells.

Supplementary Table 3 The eighteen genes and weights used to calculate the TIS score.
