# Supplementary figures and images for: Pro-inflammatory Cytokines Alter the Immunopeptidome Landscape by Modulation of HLA-B Expression
Source: Front Immunol. 2019 Feb 18;10:141. doi: 10.3389/fimmu.2019.00141 (PMC6387973; doi:10.3389/fimmu.2019.00141)

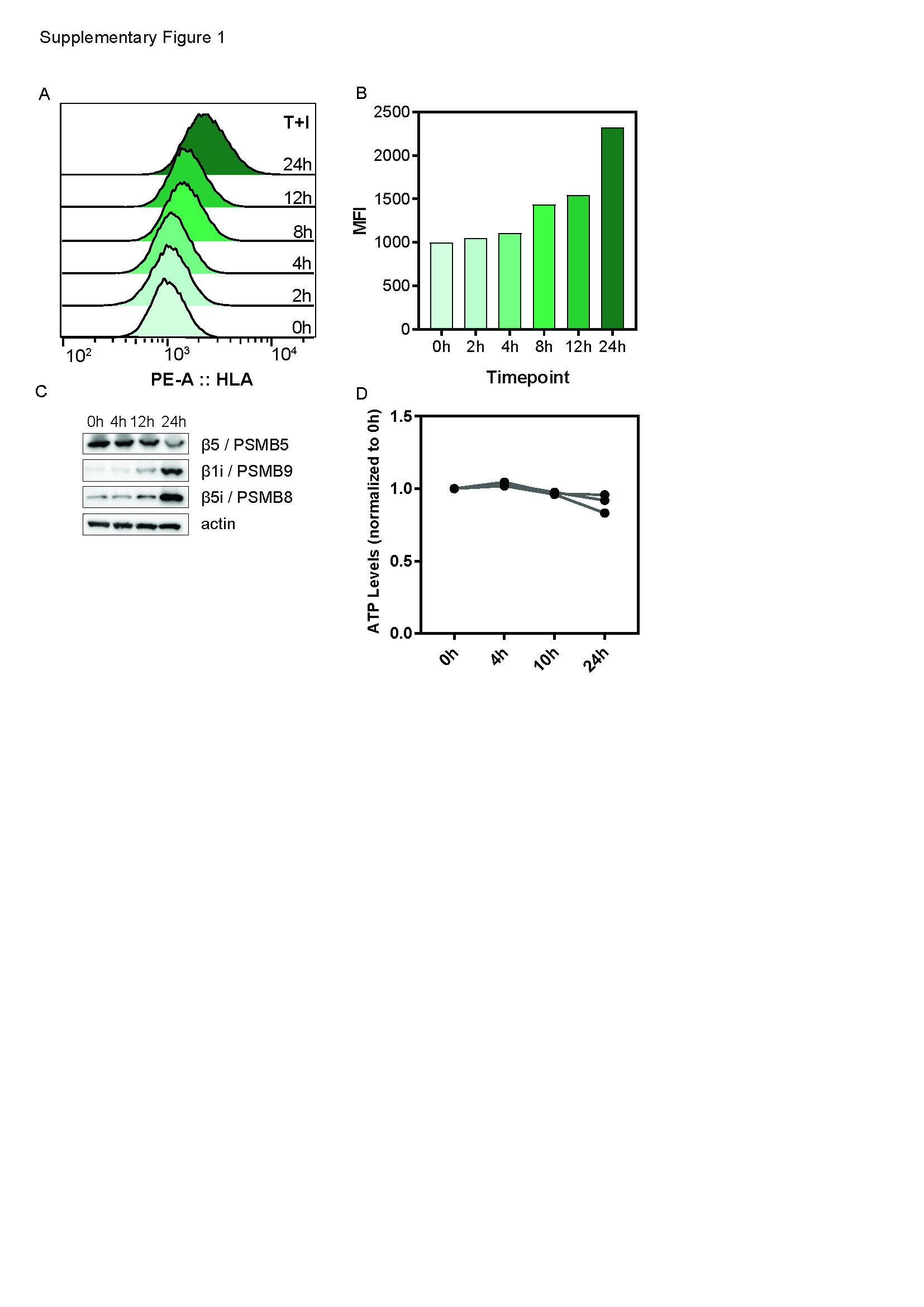

Supplement: Supplementary Figure 1 — Flow Cytometry analysis measuring the levels of HLA on the surface of A549 cells after treatment with TNFα and IFNγ for the indicated times. (A) Histograms of the fluorescence intensity corresponding to the surface expression of HLA I using PE-W6/32 (PE: HLA) and (B) the mean fluorescence intensity (MFI) are displayed. (C) Whole cell lysate from cells stimulated with TNFα and IFNγ for the indicated times was separated on SDS-PAGE and analyzed by western blot probed with the indicated antibodies. Actin is displayed as a loading control. (D) Cells stimulated with TNFα and IFNγ for the indicated times were incubated with CellTiter-Glo reagent and the measured luminescence levels were plotted (y-axis, ATP levels). Each dot represents a biological repeat of the experiment. There was no significant difference between 0 h of stimulation and 24 h (p = 0.1314). [file Image_1.JPEG]

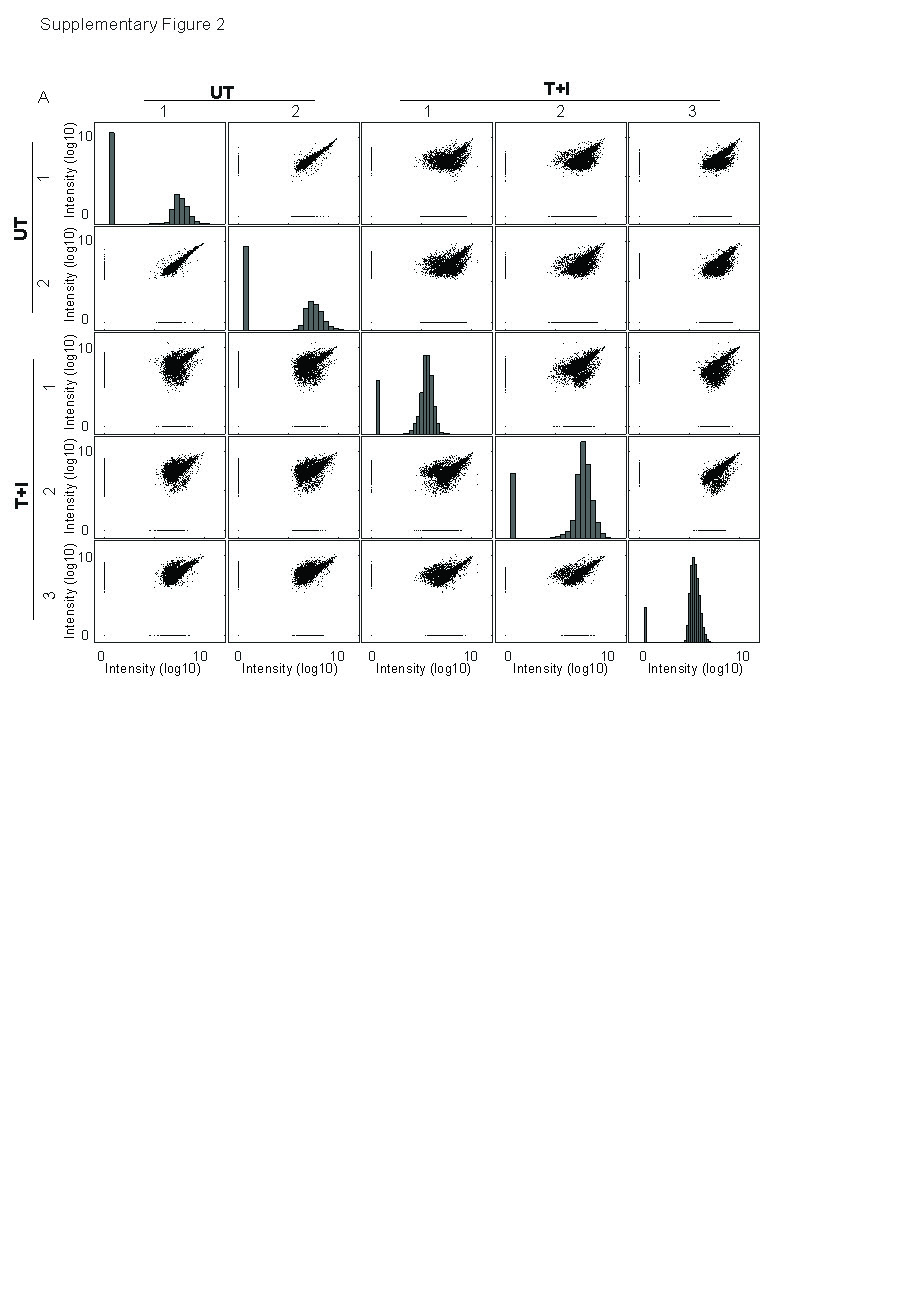

Supplement: Supplementary Figure 2 — (A) Scatter plot of the HLA presented peptide abundances (Log10) between each pairwise combination of samples. Along the diagonal of the graph, the histogram of abundance values (before imputation) for each of the samples. [file Image_2.JPEG]

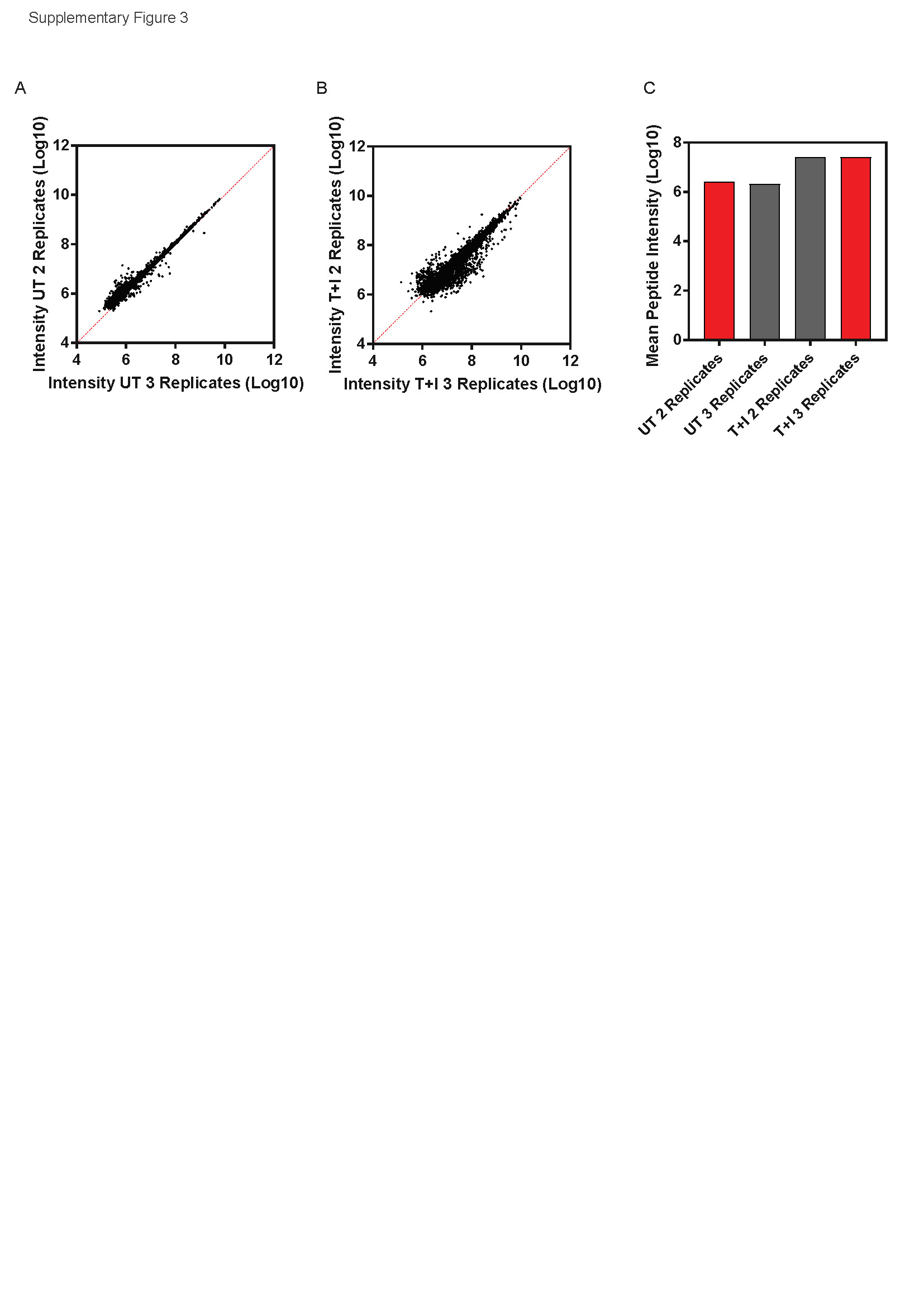

Supplement: Supplementary Figure 3 — (A,B) The median intensity of each peptide across three (x-axis) or two (y-axis) replicates from the unstimulated samples (UT; A) or TNFα and IFNγ (T+I) stimulated samples (B). A red dashed line is plotted at X = Y. (C) The mean intensity of the peptide population from two or three replicates of the unstimulated samples (UT) or TNFα and IFNγ (T+I) stimulated cells. The bars in red represent the analysis conditions chosen for the manuscript. [file Image_3.JPEG]

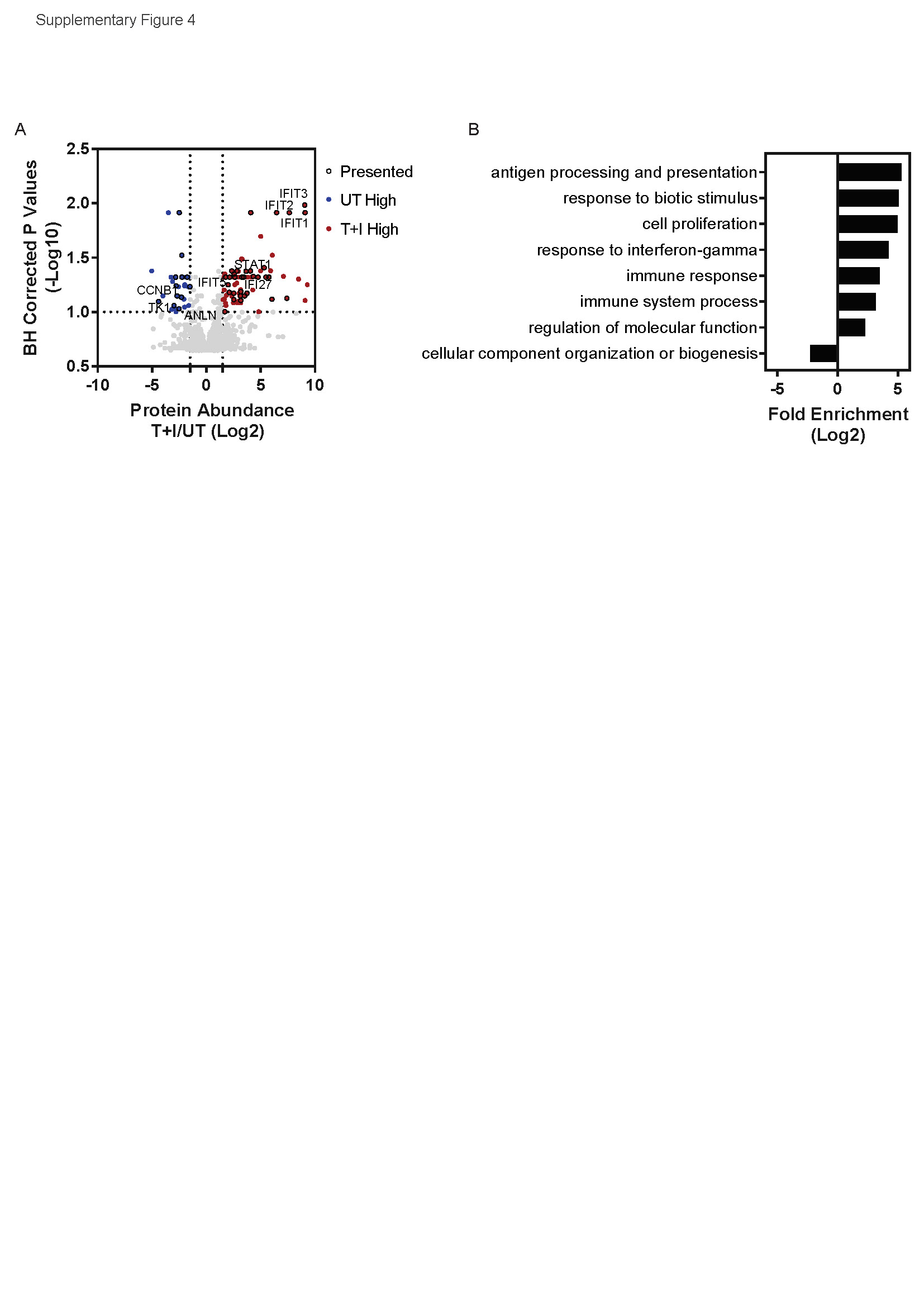

Supplement: Supplementary Figure 4 — (A) Volcano plot of the proteins identified in unstimulated cells (UT) or cells stimulated with TNFα + IFNγ (T+I). Ratios are Log2 normalized and P-values are Benjamini Hochberg FDR corrected and –Log10 transformed. Proteins enriched in T+I are marked in red, enriched in UT are marked in blue and those which contain a presented peptide are marked in a black outline. (B) Significantly enriched or underrepresented biological processes in the protein subset increased in abundance after stimulation with TNFα + IFNγ (2-fold increase or greater in abundance). All the processes passed an FDR cut-off of 0.05 and the log2 transformed normalized enrichment score is presented for each process. [file Image_4.JPEG]

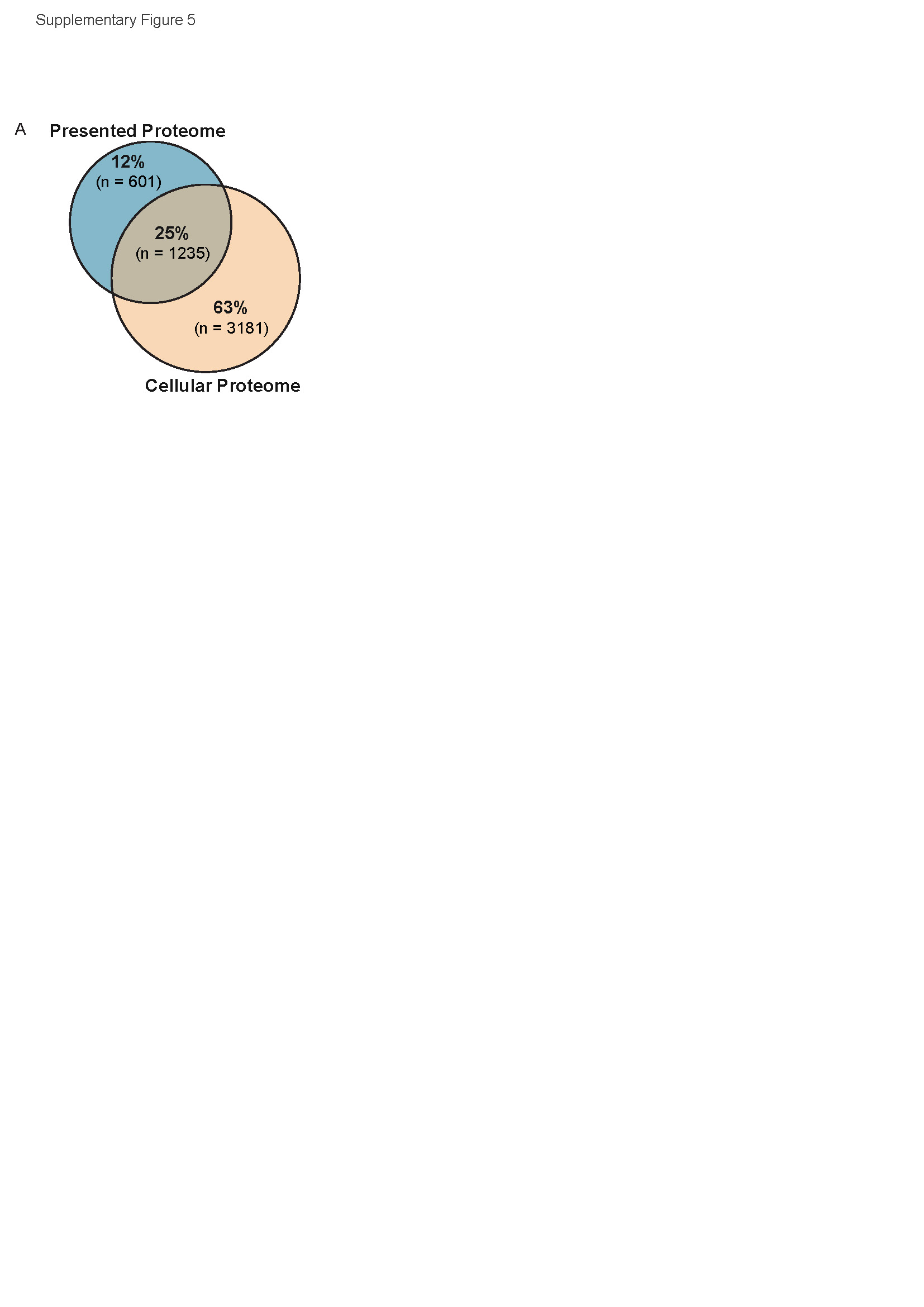

Supplement: Supplementary Figure 5 — (A) A Venn diagram showing the number and percentage of proteins identified only in the cellular proteome, only in the presented proteome or shared by both. [file Image_5.JPEG]

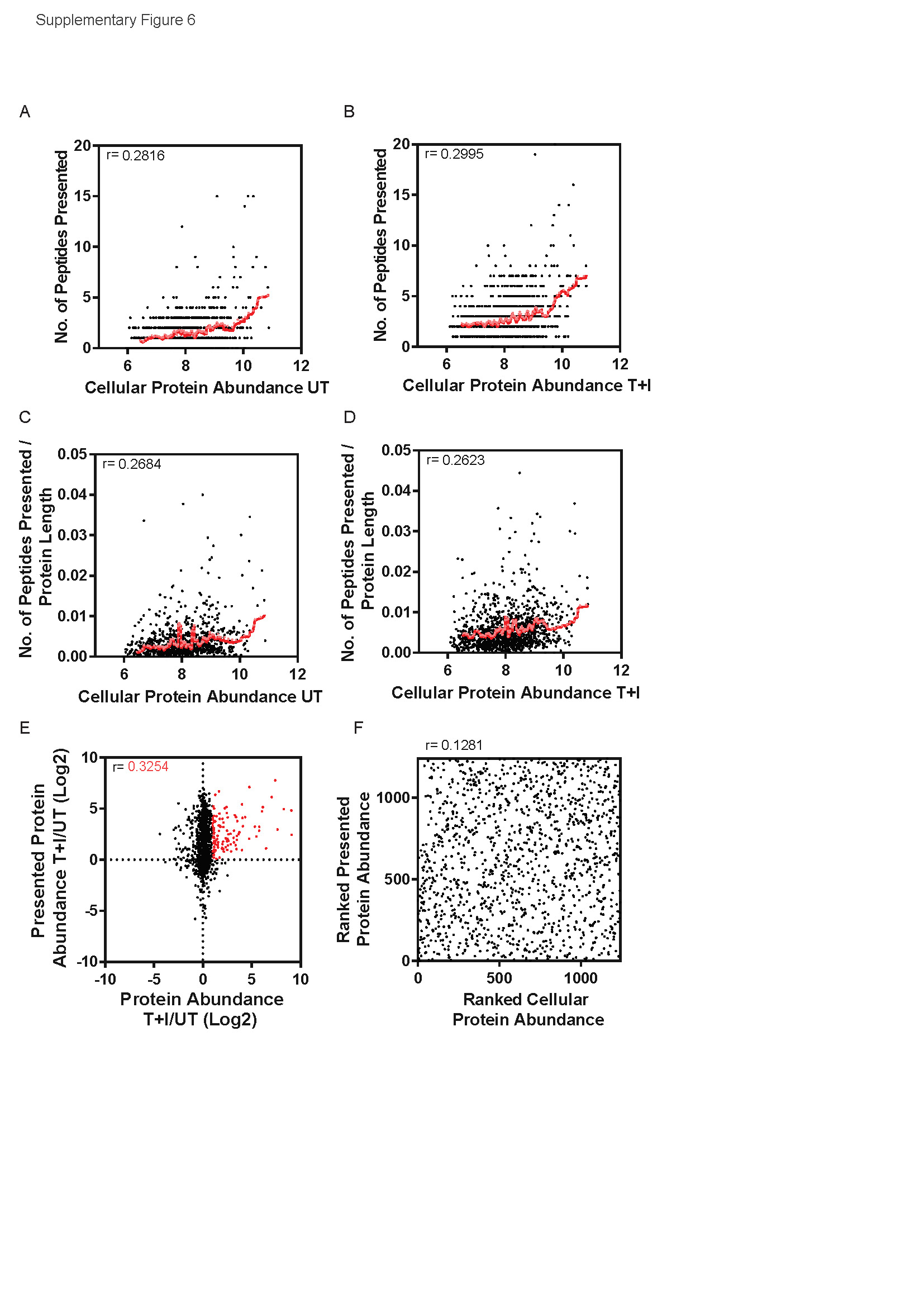

Supplement: Supplementary Figure 6 — (A,B) For either unstimulated (UT) cells (A) or cells stimulated with TNFα + IFNγ (T+I, B) the abundance of each protein in the cell was plotted against the number of unique peptides presented from that protein in stimulated cells. The red line indicates a moving average with a window of 50. Pearson correlation (r) is displayed for each plot. (C,D) For either unstimulated (UT) cells (C) or cells stimulated with TNFα + IFNγ (T+I, D) the abundance of each protein in the cell was plotted against the number of unique peptides presented from that protein and normalized to the protein length. The red line indicates a moving average with a window of 50. Pearson correlation (r) is displayed for each plot. (E) The ratio of cellular protein abundance between stimulation with TNFα + IFNγ (T+I) and unstimulated (UT) is plotted against the ratio in intensity for the presentation of each protein (Log2 transformed ratios). Proteins which increased by 2-fold or more in cellular abundance and increased in presented abundance are marked in red (n = 110). The Pearson correlation (r) for this subset is displayed on the graph (r = 0.325). (F) The ratios in Figure 3E are ranked and the ranks are plotted against one another (Log2 transformed ratios, Pearson correlation (r) is displayed on the graph, n = 1239). [file Image_6.JPEG]

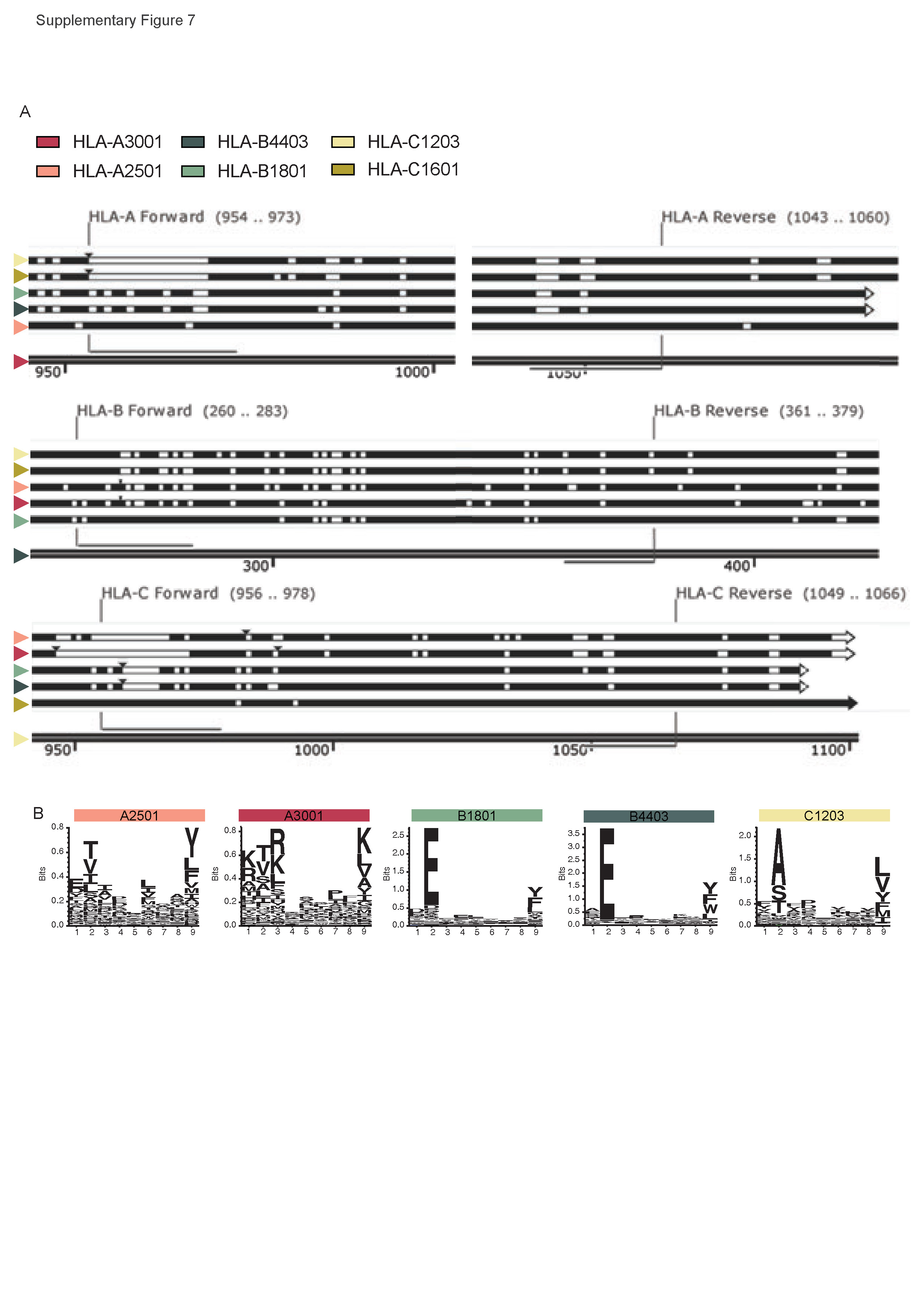

Supplement: Supplementary Figure 7 — (A) Schematic overlay of the HLA transcripts with the differential regions used to design primers. (B) The motifs of peptides known to bind to each of the five A549 haplotypes. [file Image_7.JPEG]

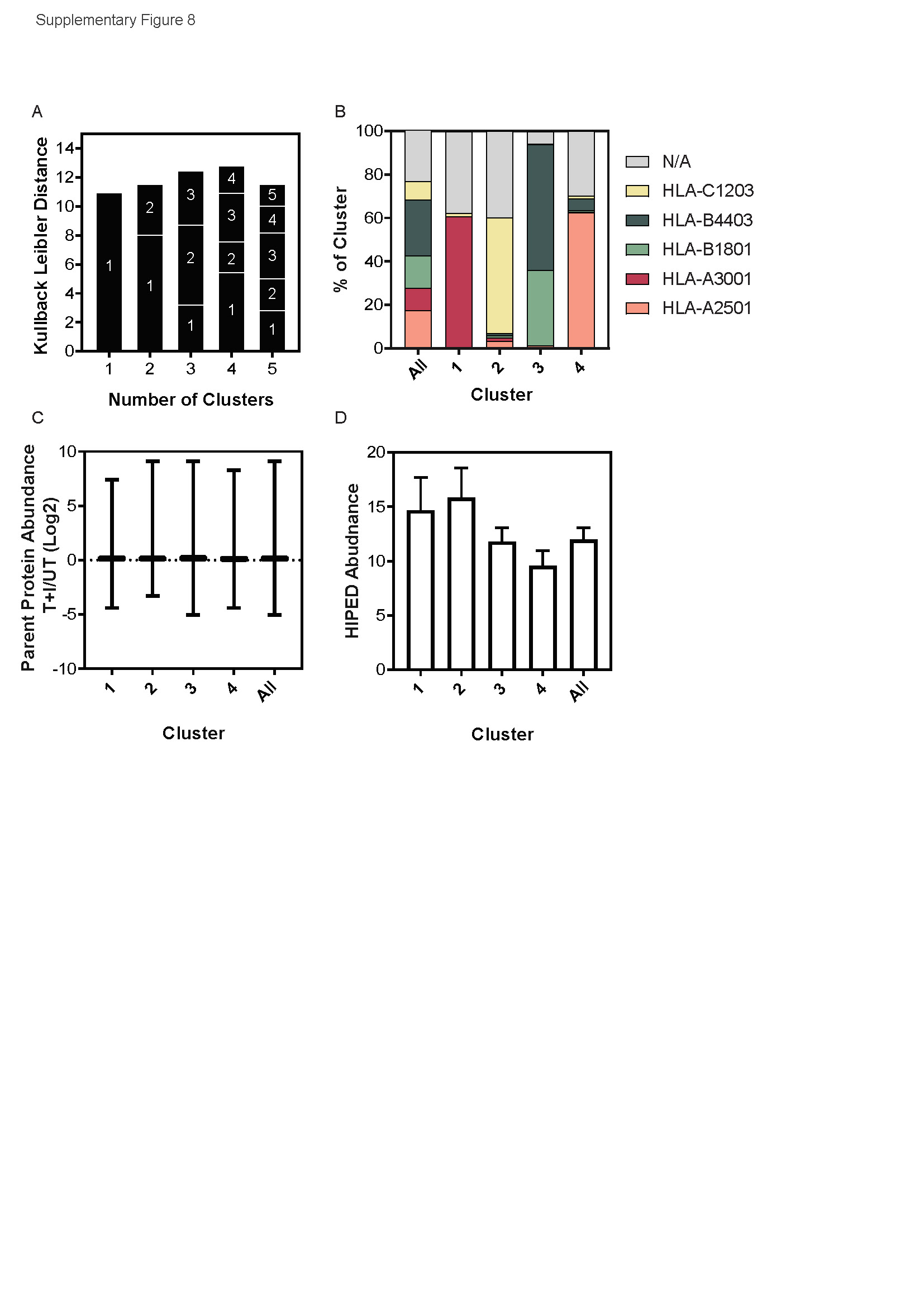

Supplement: Supplementary Figure 8 — (A) The Kullbakck-Leibler distance for Gibbs clustering performed with between 1 and 5 different clusters. (B) The percent of peptides in each cluster predicted to bind to one of the A549 haplotypes. (C) The log2 transformed ratio of protein abundance in the cell between stimulation with TNFα + IFNγ (T+I) and unstimulated (UT) for the parent proteins of the peptides in each cluster (line at median, box for the second and third quartile, lines from min to max) (D) Protein abundance was inferred from the GeneCards Suite Human Integrated Protein Expression Database (HIPED) (56) and applied to the subset of proteins in each Gibbs cluster. [file Image_8.JPEG]

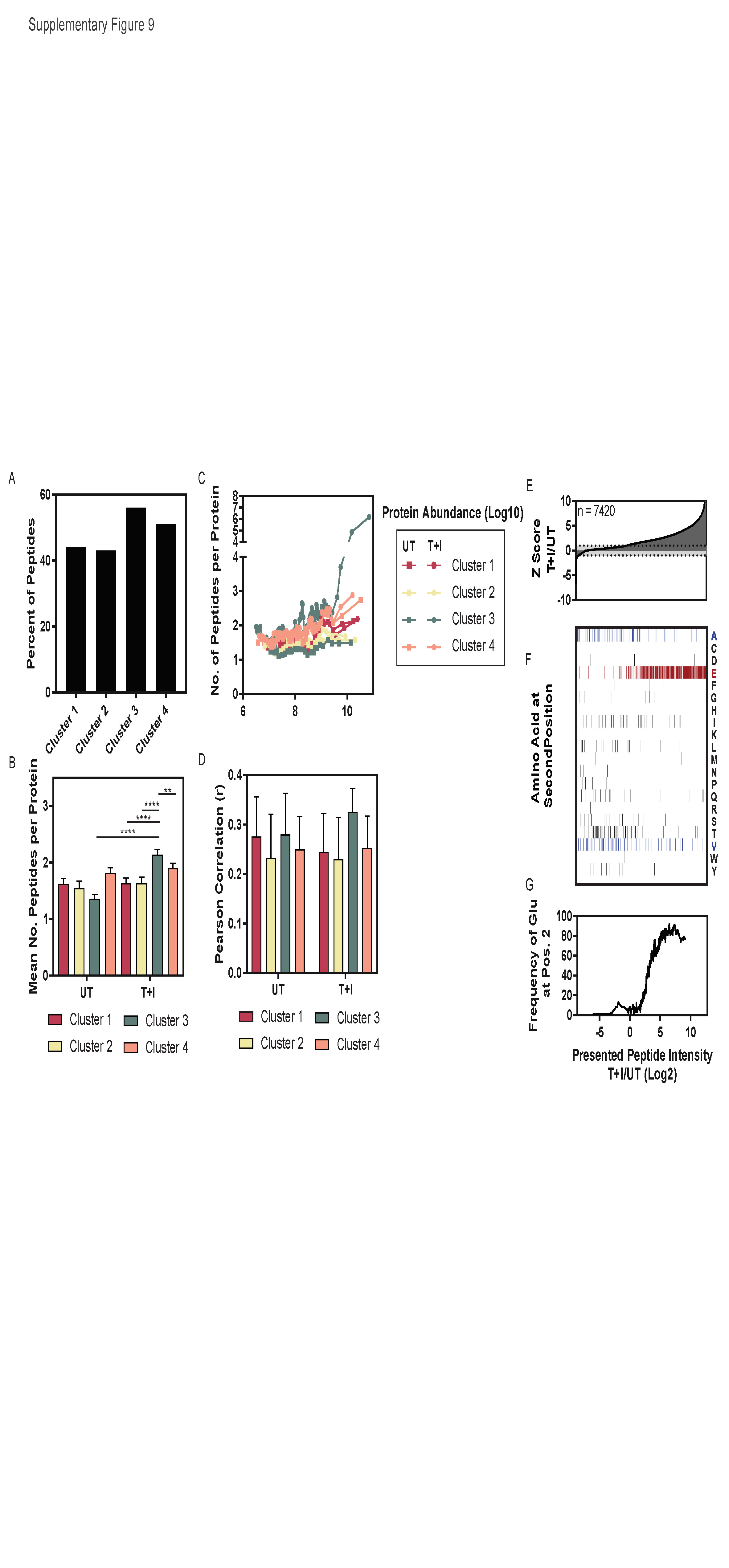

Supplement: Supplementary Figure 9 — (A) The percentage of the peptides contained in each of the 4 clusters which are peptides unique to a given protein. (B) The average number of peptides presented from a given protein (HLA sampling density) for each of the four clusters across both unstimulated (UT) or stimulated (T+I) conditions (**p = 0.0018, ****p < 0.0001; error bars indicate 95% confidence intervals on the mean). (C) The rolling average of the HLA sampling density (y axis) ranked by the abundance of the protein in the cellular lysate (x axis; rolling window = 50). Peptides were subdivided by both treatment condition and cluster. (D) The Pearson correlation between the HLA sampling density of a given protein and the abundance of that protein in the cell for each cluster and treatment combination (error bars indicate 95% confidence intervals on the correlation). (E) Each HLA presented peptide was ranked based on the change in intensity between TNFα and IFNγ stimulated and unstimulated cells (X axis is shared with G). Each protein was assigned a standard score (Z score) based on the significance of the deviation. (F) The amino acid at the second position of each peptide (X-axis is shared with G) is marked with a blue (A, V), red (E) or black (remaining amino acids). (G) The percentage of peptides which have a glutamic acid at their second position was calculated using a rolling window of 200 peptides across peptides ranked based on the change in intensity between TNFα and IFNγ stimulated and unstimulated cells. [file Image_9.JPEG]
